# Right Ventricular Strain and RV–Pulmonary Artery Coupling in Systemic Sclerosis: A Systematic Review

**DOI:** 10.3390/jcm15093368

**Published:** 2026-04-28

**Authors:** Elena Cealera, Maria-Magdalena Gurzun, Alexandra-Cristiana Gache, Monica Steluta Marc, Irinel Raluca Parepa, Elena Dantes

**Affiliations:** 1 Medical Doctoral School, Faculty of Medicine, Ovidius University of Constanta, 1 University Alley, Campus—Corp B, 900470 Constanta, Romania; pel.elena94@yahoo.com (E.C.); irinel_parepa@yahoo.com (I.R.P.); elena.dantes@365.univ-ovidius.ro (E.D.); 2 Department of Cardiology, Emergency Clinical Hospital Constanta Tomis Boulevard, 9005941 Constanta, Romania; 3 “St. Apostle Andrew” Emergency County Clinical Hospital, 900591 Constanta, Romania; 4 Department of Cardiology, University of Medicine and Pharmacy Carol Davila, 8 Eroii Sanitari Boulevard, 050474 Bucharest, Romania; magdalenagurzun@gmail.com; 5 Non-Invasive Cardio-Vascular Diseases Imaging Department, Central Emergency Military Hospital, Calea Plevnei Street No. 134, 010825 Bucharest, Romania; 6 Department of Pneumology, Faculty of Medicine, Ovidius University of Constanta, 1 University Alley, Campus-Corp B, 900470 Constanta, Romania; 7 Clinical Hospital of Pneumopthisiology Constanta, 40 Santinelei Street, 900002 Constanta, Romania; 8 Center for Research and Innovation in Precision Medicine of Respiratory Diseases, “Victor Babes” University of Medicine and Pharmacy Timisoara, Eftimie Murgu Square 2, 300041 Timisoara, Romania; stel_marc@yahoo.com; 9 Discipline of Pulmonology, “Victor Babes” University of Medicine and Pharmacy Timisoara, 300041 Timisoara, Romania

**Keywords:** systemic sclerosis, interstitial lung disease, right ventricular strain, RV–pulmonary artery coupling, NT-proBNP, speckle-tracking echocardiography, pulmonary hypertension

## Abstract

**Background**: Right ventricular (RV) dysfunction is a key contributor to morbidity and mortality in systemic sclerosis (SSc), emerging from the combined effects of microvascular disease, myocardial fibrosis, interstitial lung involvement, and increasing pulmonary vascular load. Conventional echocardiography frequently fails to detect early RV impairment, prompting growing interest in deformation-based parameters such as RV free-wall longitudinal strain (RV-FWS), global longitudinal strain (RV-GLS), and RV–pulmonary artery (PA) coupling indices. Although natriuretic peptides reflect myocardial stress and are widely used in cardiopulmonary diseases, their integration with advanced RV imaging has been inconsistently reported in SSc. This systematic review synthesizes available evidence on RV strain, RV–PA coupling, and their relationship with clinical outcomes and biomarkers in SSc. **Methods**: A systematic search was conducted to identify clinical studies evaluating RV strain (RV-FWS, RV-GLS), right atrial strain, or RV–PA coupling indices in adult patients with SSc or SSc-associated pulmonary arterial hypertension (SSc-PAH). Eligible studies included those using speckle-tracking echocardiography or cardiac magnetic resonance feature-tracking. Study selection and data extraction were performed in accordance with PRISMA guidelines. **Results**: Seven studies met the eligibility criteria. Across unselected SSc cohorts, early disease without pulmonary hypertension (PH), and right-heart-catheterization-confirmed SSc-PAH, RV strain consistently detected myocardial impairment even when conventional echocardiographic indices remained normal. RV-FWS and RV-GLS were commonly reduced, and longitudinal data demonstrated progressive deterioration independent of standard measures. Strain-derived RV–PA coupling, particularly RV-FWS/PASP, significantly improved prognostic stratification when added to established PAH risk models. Two studies identified impaired RV deformation as a predictor of mortality, and CMR-derived right atrial strain provided additional prognostic value. Biomarker integration was limited, with only one study reporting an association between natriuretic peptide elevation (NT-proBNP) and impaired RV–PA coupling suggesting that biomarkers may reflect the hemodynamic load, although evidence remains limited captured by strain abnormalities. **Conclusions**: RV strain and RV–PA coupling indices are more sensitive than conventional echocardiography for detecting early RV dysfunction, monitoring disease progression, and predicting adverse outcomes in SSc. Although biomarker evidence remains limited, available data suggest that natriuretic peptides may provide complementary information to deformation-based assessment, although current evidence remains limited by reflecting combined myocardial and pulmonary vascular load. Standardized prospective studies including both strain imaging and biomarkers are needed to clarify the integrated diagnostic and prognostic value of advanced RV assessment in SSc.

## 1. Introduction

Systemic sclerosis (SSc) is a heterogeneous autoimmune disease characterized by microvascular dysfunction, inflammation, and progressive fibrosis affecting multiple organ systems [1,2,3]. SSc is also increasingly recognized as a condition associated with a substantially elevated risk of cardiovascular mortality. Recent evidence from a systematic review and meta-analysis by Figliozzi et al., indicates that SSc is among the systemic immune-mediated diseases most strongly associated with cardiovascular death, with a risk profile largely driven by inflammation-mediated mechanisms, particularly an increased incidence of acute coronary syndromes [4]. This expanded cardiovascular risk profile reflects the complex interplay between macrovascular disease, microvascular dysfunction, and myocardial involvement, which collectively contribute to both left- and right-sided cardiac impairment. In this context, right ventricular (RV) dysfunction should be interpreted as part of a broader cardiovascular continuum rather than an isolated consequence of pulmonary vascular disease, highlighting the need for integrated cardiopulmonary assessment strategies.

Within this framework, cardiopulmonary involvement emerges as a key determinant of clinical outcomes in SSc. Within this broader cardiovascular context, cardiopulmonary involvement represents a key interface between systemic inflammation and organ-specific complications in SSc. Cardiopulmonary involvement is common and represents a major driver of morbidity and mortality. Among these complications, right ventricular (RV) dysfunction plays a central prognostic role, reflecting the combined burden of pulmonary vascular disease, myocardial fibrosis, and interstitial lung involvement. Early recognition of RV impairment is therefore crucial, yet conventional echocardiographic indices such as tricuspid annular plane systolic excursion (TAPSE), fractional area change (FAC), or right-sided chamber dimensions often fail to detect subtle abnormalities in myocardial performance [5,6,7].

Deformation imaging has emerged as a more sensitive method for assessing RV function. Speckle-tracking echocardiography enables quantification of RV free-wall longitudinal strain (RV-FWS) and global longitudinal strain (RV-GLS), while cardiac magnetic resonance (CMR) feature-tracking provides complementary insights into RV and right atrial (RA) mechanics [8,9,10,11]. Across different SSc phenotypes—including early disease without pulmonary hypertension (PH), unselected SSc cohorts, and patients with right-heart-catheterization-confirmed SSc-associated pulmonary arterial hypertension (SSc-PAH)—RV strain abnormalities have been reported even when standard echocardiographic measures remain within normal limits [9,12,13]. These findings suggest that strain may detect early myocardial involvement before overt hemodynamic or structural changes become apparent.

An additional area of interest is the assessment of RV–pulmonary artery (PA) coupling, which reflects the interaction between RV contractility and pulmonary arterial load [14,15]. Strain-derived coupling indices, such as RV-FWS/PASP, have shown promise in refining prognostic evaluation, particularly in SSc-PAH. CMR-derived RA strain parameters have also been identified as independent predictors of adverse outcomes [7,16], highlighting the potential of advanced deformation analysis in risk stratification.

Although natriuretic peptides are well-established markers of myocardial stress, their integration with advanced imaging metrics remains limited, with sparse and heterogeneous data directly linking NT-proBNP to strain-derived parameters. To date, only one study has explored this relationship, suggesting a potential association with RV–pulmonary artery coupling; however, evidence remains insufficient to support definitive conclusions [16]. These findings raise the possibility that combining biomarkers with advanced imaging may enhance phenotyping of cardiopulmonary involvement in SSc [17,18,19].

Given the variability in methodologies, patient populations, and imaging parameters across published studies, a synthesis of existing evidence is needed to clarify the diagnostic and prognostic utility of RV strain and RV–PA coupling in SSc [7,9,10,12,13]. This systematic review examines seven studies employing echocardiographic speckle-tracking or CMR feature-tracking to evaluate RV deformation, RA strain, and RV–PA coupling in SSc, to establish their clinical importance and identify unresolved gaps in the literature.

## 2. Materials and Methods

This systematic review was conducted in accordance with the Preferred Reporting Items for Systematic Reviews and Meta-Analyses (PRISMA) guidelines, as detailed in Table S1 an internationally recognized and widely adopted methodological framework [20]. The review was prospectively registered in the PROSPERO database (International Prospective Register of Systematic Reviews) under the registration number CRD420251235679.

### 2.1. Search Strategy

A comprehensive literature search was performed in three major electronic databases: PubMed, Web of Science, and ScienceDirect (Elsevier). The search included all available records published up to October 2025, restricted to human studies, full-text articles, and English-language publications, using the following Boolean expression: (“systemic sclerosis” OR “scleroderma”) AND (“right ventricular strain” OR “RV strain” OR “speckle tracking”) AND (“pulmonary hypertension” OR “interstitial lung disease” OR “NT-proBNP”) (Table 1).

The research question guiding this review was structured according to the Patient/Problem, Intervention, Comparison and Outcome framework (PICO), as follows: In patients with SSc (P), how does the assessment of right ventricular function using speckle-tracking-derived strain parameters (I), compared with conventional echocardiographic indices or the absence of strain evaluation (C), relate to the detection, severity, and clinical significance of cardiopulmonary involvement—particularly interstitial lung disease, pulmonary hypertension, and biomarker elevation such as NT-proBNP (O)?

### 2.2. Eligibility Criteria

Studies were considered eligible for inclusion if they fulfilled the following criteria: they were published in English, available in full-text form as open-access publications or obtained directly from the authors, and included adult participants (≥18 years) with a confirmed diagnosis of SSc according to established classification standards. Eligible studies were required to evaluate right ventricular systolic function using speckle-tracking echocardiography or an equivalent strain-based imaging technique, and to report at least one advanced deformation parameter such as right ventricular global longitudinal strain (RV-GLS), right ventricular free-wall longitudinal strain (RV-FWS), or indices reflecting RV–pulmonary artery coupling [7,9,10,12,13]. Furthermore, studies had to provide clinically relevant comparative data, including conventional echocardiographic indices (e.g., TAPSE, FAC), measures of interstitial lung disease severity, pulmonary hypertension status, biomarker profiles such as NT-proBNP, or functional and hemodynamic correlates including DLCO, FVC, HRCT fibrosis extent, or prognostic outcomes.

Studies were excluded if they involved younger than 18 years, mixed connective tissue diseases or other autoimmune diseases without separately reported SSc-specific data consisted of case reports, conference abstracts without data, narrative reviews, editorials, or duplicate datasets. Additionally, articles that did not analyze SSc patients separately within mixed connective-tissue disease cohorts, studies lacking quantitative RV strain metrics (only TAPSE/FAC/S′/dimensions), studies assessing cardiac function in SSc but not evaluating strain or RV–PA coupling or were unavailable in full text were excluded from the final selection.

One study was excluded from the final synthesis. Although it provided valuable biomarker data relevant to cardiopulmonary involvement in SSc, it did not include quantitative right ventricular strain assessment using speckle-tracking echocardiography or cardiac magnetic resonance feature-tracking techniques. As a result, it did not meet the predefined inclusion criteria, which required the evaluation of deformation-based RV parameters and/or RV–pulmonary artery coupling indices.

### 2.3. Data Extraction

Citations retrieved from PubMed, ScienceDirect (Elsevier) and Web of Science, were imported into Zotero for management. Duplicates were identified and removed after exporting the library to Microsoft Excel, using the “remove duplicates” function.

### 2.4. Risk of Bias Assessment

The risk of bias for the included studies was assessed using the ROBINS-I V2 tool (Risk of Bias in Non-randomized Studies of Interventions, Version 2), specifically developed for the evaluation of non-randomized observational research. This instrument enables a structured and comprehensive appraisal of potential bias across seven methodological domains: bias due to confounding; bias in the classification of interventions or exposures; bias in participant selection; bias arising from deviations from intended interventions; bias due to missing data; bias in outcome measurement; and bias in the selection of reported results.

This assessment was conducted independently by two authors (E.D. and E.C.), with disagreements resolved through discussion and consensus among all authors (E.C., M.-M.G., A.-C.G., M.S.M., I.R.P., and E.D.). This systematic and transparent approach ensured a consistent and rigorous evaluation of internal validity and methodological robustness across the studies included in this review.

## 3. Results

### 3.1. Search

A total of 430 records were identified through database searching, including PubMed (*n* = 18), Web of Science (*n* = 78), and ScienceDirect (*n* = 338), by E.C., M.-M.G., and A.-C.G. After the removal of 22 duplicate entries by E.C. and E.D., 408 unique citations were retained for initial screening.

Titles and abstracts were independently screened by two reviewers (E.C. and A.-C.G.) to assess their relevance with respect to the predefined eligibility criteria, leading to the exclusion of 296 articles that were clearly unrelated to SSc, did not evaluate right ventricular strain, or did not report relevant clinical or biomarker outcomes. The screening strategy and eligibility criteria were reviewed and validated by M.-M.G. and I.R.P.

The remaining 112 records were selected for abstract review. This stage was conducted by E.C. and M.S.M., with methodological oversight provided by E.D. Of these, 77 were excluded because they did not meet the eligibility criteria, due to their lack of alignment with the central research question, absence of relevant methodology, or focus on unrelated populations or diagnostic techniques.

A total of 35 full-text articles were retrieved for eligibility assessment by A.-C.G. and I.R.P. Following a comprehensive review, studies were excluded if they did not provide quantitative strain parameters, failed to report NT-proBNP or pulmonary hypertension outcomes, lacked extractable SSc subgroup data, or did not align with the central research objectives. Discrepancies were resolved through consensus with E.C. and E.D.

Ultimately, 7 studies met all inclusion criteria and were incorporated into the qualitative synthesis. The final inclusion decision was agreed upon by all authors (E.C., M.-M.G., A.-C.G., M.S.M., I.R.P., and E.D.).

These seven studies are summarized in Table 2, which includes information on authorship, year of publication, study design, SSc subtype, imaging methodology, strain parameters evaluated, and key clinical outcomes. Data extraction and table construction were performed by E.C. and A.-C.G.

This systematic review demonstrates that right ventricular (RV) deformation imaging provides important diagnostic and prognostic value in SSc. Across heterogeneous cohorts—including patients without pulmonary hypertension (PH), unselected SSc populations, and individuals with confirmed SSc–associated pulmonary arterial hypertension (SSc-PAH)—RV free-wall strain (RV-FWS), global longitudinal strain (RV-GLS), and strain-derived RV–pulmonary artery (PA) coupling indices consistently identified myocardial involvement earlier and more reliably than conventional echocardiographic parameters. These findings highlight the unique vulnerability of the RV in SSc, driven by the combined effects of microvascular dysfunction, myocardial fibrosis, and variable pulmonary vascular loading [21,22].

Cardiac magnetic resonance (CMR) feature tracking further complements echocardiography by providing additional insights into RV mechanics, right atrial (RA) function, and myocardial tissue characteristics, all of which demonstrated prognostic relevance in the included studies [7,16]. Preliminary biomarker data suggest a potential but still limited role for natriuretic peptides in reflecting RV–PA maladaptation, although evidence remains limited and requires further investigation.

Collectively, the available literature supports the integration of RV strain and RV–PA coupling into the cardiopulmonary assessment of SSc, particularly in patients with early symptoms, borderline hemodynamics, or discordant clinical findings [8,14,15]. However, methodological heterogeneity, limited longitudinal data, and inconsistent biomarker integration underline the need for larger, prospective studies using standardized imaging protocols [11]. Advancing a multimodal approach that combines deformation imaging, CMR metrics, pulmonary function measures, and biomarkers may improve early detection, phenotyping, and risk stratification in this complex, multisystem disease [21,22].

**Figure 1:**
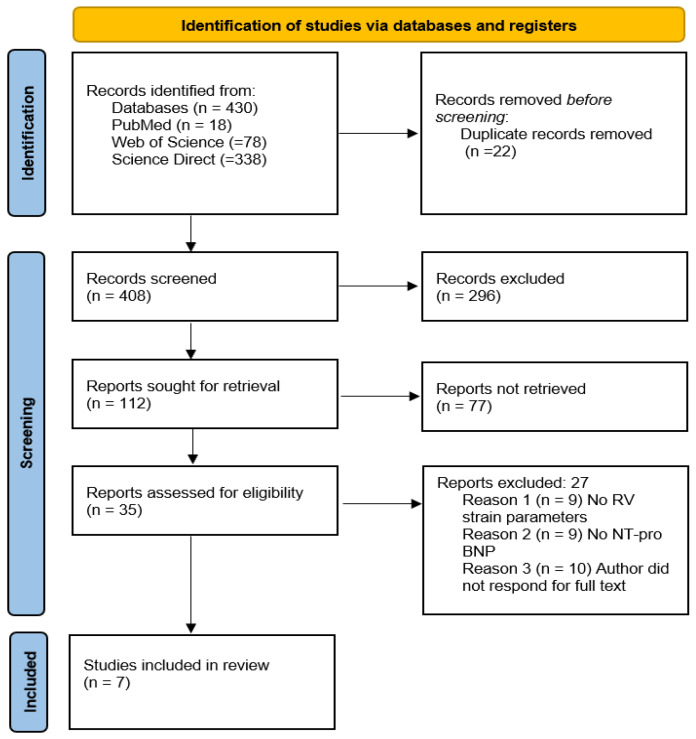
Our adapted PRISMA flow diagram outlines the process of study identification, screening, eligibility assessment and inclusion.

Right ventricular systolic function in individuals with SSc using speckle-tracking-derived strain imaging, either independently or in conjunction with conventional echocardiographic indices and cardiopulmonary markers (Figure 1).

**Table 2:** Summary of study characteristics and principal findings from the seven included investigations evaluating right ventricular strain and RV–pulmonary artery coupling in SSc. The table reports study design, population characteristics, imaging techniques used, and key clinical associations with strain parameters, ILD severity, pulmonary vascular status, and NT-proBNP [1,5,6,7,23,24,25].

Authors	Year	Study Design	Population/SSc Subtype	Technique(s)	Main Findings
Hekimsoy et al. [5]	2018	Prospective observational	80 SSc patients	TTE + 2D STE (regional RV-FWS)	Apical RV-FWS predicted PAH; regional RV strain declined at 12-month follow-up; RA area and TRV were strong PH predictors. NT-proBNP not included.
Tennøe et al. [1]	2019	Prospective cohort	277 SSc patients	TTE (RV strain, TAPSE)	Progressive RV dysfunction predicted mortality; TAPSE decline clinically relevant. NT-proBNP measured but no correlation with strain reported.
Stronati et al. [6]	2020	Prospective longitudinal (20 months)	72 SSc without PH	TTE + STE (LV/RV GLS, layer-specific strain)	RV and LV GLS progressively deteriorated over 20 months; worsening GLS predicted PH, MACE and mortality. NT-proBNP measured but not analyzed vs. strain.
Vos et al. [7]	2022	Retrospective cohort	100 SSc patients (limited & diffuse)	CMR feature tracking (RA/RV strain)	RA reservoir and conduit strain predicted mortality; RV strain not independently prognostic. NT-proBNP included descriptively no strain comparison.
Fernández et al. [23]	2024	Retrospective cohort	91 SSc patients (67% lcSSc; 33% dcSSc)	CMR feature tracking (RV strain, RV volumes, RVEF; LGE assessment)	RVD prevalence 35%. Patients with RVD had significantly worse RV strain (−15.4% vs. −25%), larger RV volumes, more LGE, and higher BNP levels.
Lui et al. [25]	2025	Retrospective cohort	124 SSc-PAH patients	TTE + STE (LV GLS, RV-FWS), RHC	LV strain clusters predicted impaired RV-FWS and higher mortality. NT-proBNP measured but not analyzed in relation to Strain.
Osgueritchian et al. [24]	2025	Retrospective observational	174 SSc-PAH patients	TTE (RV-GLS, RV-FWS, RV-PA coupling via RV-GLS/PASP, RV-FWS/PASP) + risk scores	Adding RV-PA coupling metrics significantly improved 1-yr mortality prediction across COMPERA, REVEAL, and French criteria.

Abbreviations: SSc, SSc; PH, pulmonary hypertension; PAH, pulmonary arterial hypertension; ILD, interstitial lung disease; TTE, transthoracic echocardiography; STE, speckle-tracking echocardiography; 2D STE, two-dimensional speckle-tracking echocardiography; CMR, cardiac magnetic resonance; RHC, right heart catheterization; RV, right ventricle; LV, left ventricle; RA, right atrium; RV-FWS, right ventricular free-wall strain; RV-GLS, right ventricular global longitudinal strain; LV GLS, left ventricular global longitudinal strain; TRV, tricuspid regurgitant velocity; TAPSE, tricuspid annular plane systolic excursion; NT-proBNP, N-terminal pro-B-type natriuretic peptide; MACE, major adverse cardiovascular events.

### 3.2. Description of Selected Studies

The seven included studies comprised heterogeneous SSc populations encompassing unselected cohorts, patients without pulmonary hypertension (PH), and individuals with right-heart-catheterization (RHC)-confirmed SSc-associated pulmonary arterial hypertension (SSc-PAH). Study sizes ranged from 72 to 277 participants, with varying distributions of limited and diffuse cutaneous SSc. Five investigations used transthoracic echocardiography with speckle-tracking analysis, and two studies employed cardiac magnetic resonance (CMR) feature-tracking to evaluate right ventricular (RV) and right atrial (RA) mechanics. A detailed overview is presented in Table 2.

Three echocardiographic studies evaluated SSc patients without established PH. Hekimsoy et al. (2018) analyzed regional RV free-wall strain (RV-FWS) in 80 SSc patients and demonstrated that apical RV-FWS impairment predicted incident PAH, while regional strain deterioration occurred over 12-month follow-up [5]. Tennøe et al. (2019) examined 277 patients and reported that progressive RV dysfunction and TAPSE decline were associated with increased mortality [1]. Stronati et al. (2020) followed 72 SSc patients without PH over 20 months and found that both RV and LV global longitudinal strain (GLS) progressively worsened despite preserved conventional echocardiographic parameters, with GLS deterioration predicting PH development, major adverse cardiac events (MACE), and mortality [6].

Two studies investigated RV and RA deformation using CMR. Vos et al. assessed RA and RV strain in 100 SSc patients and identified RA reservoir and conduit strain as independent predictors of mortality, whereas RV strain was not associated with outcomes after multivariable adjustment [7]. Fernández et al. evaluated 91 SSc patients and reported a 35% prevalence of RV dysfunction (RVD). Patients with RVD demonstrated significantly impaired right-ventricular longitudinal strain increased late gadolinium enhancement (LGE), higher BNP levels, and significantly greater risk of heart failure hospitalization and mortality [23].

Three studies focused specifically on SSc-PAH. Osgueritchian et al. (2025) included 174 RHC-confirmed SSc-PAH patients and demonstrated that strain-derived RV–PA coupling indices (RV-FWS/PASP, RV-GLS/PASP) significantly improved 1-year mortality prediction when integrated into established prognostic models such as COMPERA, REVEAL, and the French non-invasive criteria [24]. Lui et al. (2025) evaluated 124 SSc-PAH patients and reported that distinct LV deformation phenotypes were strongly associated with impaired RV-FWS and poorer survival, underscoring the prognostic importance of biventricular strain analysis in advanced disease [25].

Although not included in the final synthesis because it did not assess RV strain parameters, the study by Grimaldi et al. (2023) provides complementary biological insights: elevated natriuretic peptide levels (NT-proBNP) were associated with impaired TAPSE/sPAP and contributed to improved early identification of pulmonary vascular involvement. This highlights the potential value of integrating biomarker information alongside advanced imaging findings [26].

Overall, the selected studies consistently indicated that RV strain and strain-derived RV–PA coupling provide enhanced sensitivity for detecting early myocardial involvement and improved prognostic stratification across the SSc spectrum.

### 3.3. Risk of Bias

**Figure 2:**
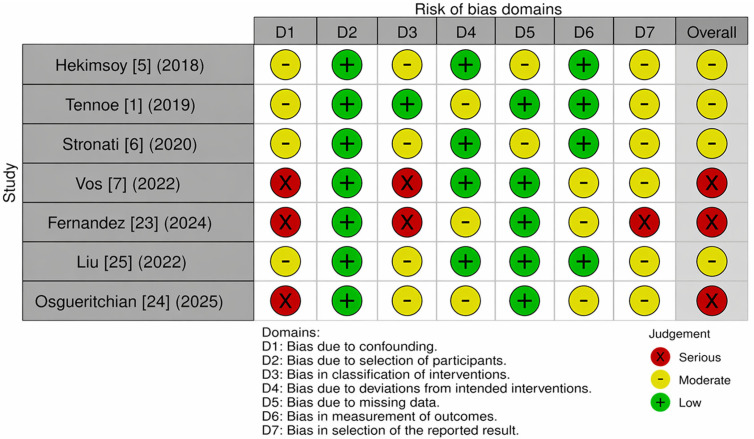
Summary of included studies evaluating right ventricular strain and RV–pulmonary artery coupling in SSc, including Hekimsoy et al. [5], Tennøe et al. [1], Stronati et al. [6], Vos et al. [7], Fernández et al. [23], Lui et al. [25], and Osgueritchian et al. [24].

Across the seven included studies, the overall risk of bias is illustrated in Figure 2 ranged from moderate to serious, with the majority of studies (4/7) judged as having a moderate overall risk and three studies were rated as serious. The domains most frequently affected were confounding, missing data, and selection of the reported result.

Overall, the risk of bias assessment revealed considerable methodological variability among the included studies, which must be taken into account when interpreting the diagnostic and prognostic value of right ventricular (RV) strain and RV–pulmonary artery (PA) coupling in SSc. Most concerns were concentrated in domains related to confounding, participant classification, and selective reporting, whereas the technical domains—particularly measurement of outcomes—were consistently robust.

Bias due to confounding (D1) was the most critical limitation, especially in studies by Vos et al., Fernandez et al., and Osgueritchian et al., which were rated at serious risk. These cohorts included patients with advanced cardiopulmonary involvement, heterogeneous ILD load, or variable PAH therapies, without adequate statistical adjustment. As a result, the observed associations between strain abnormalities and outcomes may partially reflect underlying disease severity rather than intrinsic myocardial dysfunction [6,23,24]. In contrast, studies with low-to-moderate risk of confounding [1,5,6,25] provide more reliable evidence for the role of strain in early disease detection, as they enrolled more homogeneous populations and applied standardized assessment protocols.

Bias in classification of participants and interventions (D2–D3) was another important issue. Studies based on clinically indicated CMR examinations inherently selected patients with suspected or established cardiac disease, introducing a severity-selection bias that limits generalizability. This was particularly relevant for Vos et al. and Fernandez et al., where the likelihood of detecting strain abnormalities was already high due to the pre-selected population. In contrast, echocardiographic screening cohorts demonstrated lower risk in these domains.

Bias due to missing data (D5) was generally low to moderate across studies, with limited attrition and acceptable handling of incomplete measurements. However, retrospective designs occasionally lacked complete biomarker data or uniform follow-up, preventing integrated analyses of strain and natriuretic peptides.

The measurement of outcomes (D6) was consistently the strongest methodological component. All studies used validated software for speckle-tracking or CMR feature tracking, followed standardized acquisition protocols, and reported interobserver reproducibility where applicable. This supports the reliability of the imaging-derived parameters themselves, even when other methodological aspects were suboptimal.

Selective reporting (D7) emerged as a notable limitation in studies with serious overall bias. The lack of prespecified protocols and the preferential reporting of statistically significant strain parameters raise the possibility of reporting bias, which may have amplified the apparent prognostic performance of certain indices, particularly right atrial strain in CMR-based studies.

Overall, while the technical quality of imaging measurements was high, the presence of confounding and selection biases in several studies indicates that the magnitude of association between RV strain, RV–PA coupling, and clinical outcomes should be interpreted cautiously. Nevertheless, the consistency of findings across studies with varying risk of bias supports the role of strain-based assessment as a sensitive tool, although future research should incorporate standardized methodologies and robust adjustment strategies to confirm its diagnostic and prognostic utility.

## 4. Discussions

SSc is a complex autoimmune disorder characterized by immune-mediated endothelial injury, microvascular dysfunction, and progressive fibrosis affecting the skin and multiple internal organs. Among its visceral complications, cardiopulmonary involvement remains a major determinant of morbidity and mortality, largely driven by interstitial lung disease (ILD) and pulmonary arterial hypertension (PAH) [27,28,29]. Although clinical manifestations often appear late in the disease course, accumulating evidence demonstrates that subclinical cardiac impairment—particularly right ventricular (RV) dysfunction—occurs early and may precede hemodynamic deterioration by several years [9,12,15].

Myocardial deformation imaging has emerged as a sensitive and reproducible method for detecting these early abnormalities [8,11]. Unlike conventional echocardiographic parameters such as TAPSE or FAC, which are substantially influenced by loading conditions and may remain within normal limits despite evolving myocardial dysfunction, speckle-tracking-derived RV strain provides a more refined assessment of longitudinal fiber shortening, the primary contributor to RV systolic performance. Across multiple cohorts, impaired RV free-wall strain (RV-FWS) and global longitudinal strain (RV-GLS) have been observed in SSc patients even in the absence of overt PH, suggesting intrinsic myocardial involvement [9,12], microvascular ischemia, or subtle increases in pulmonary vascular resistance that are not captured by standard hemodynamic thresholds.

Despite the widespread clinical use of N-terminal pro-brain natriuretic peptide (NT-proBNP) as a biomarker of myocardial stress, its integration with advanced imaging metrics remains insufficiently explored, with limited and heterogeneous evidence currently available across published studies [16,17,18]. As a result, the biochemical–mechanical interplay underlying RV dysfunction in SSc is not yet fully characterized. Understanding this relationship is essential for improving risk stratification, refining early detection of pulmonary vascular disease, and strengthening the prognostic framework for patients with SSc. Integrating deformation imaging with biomarkers such as NT-proBNP, and contextualizing these findings within the broader spectrum of ILD severity and pulmonary function impairment, may provide a more precise representation of cardiopulmonary burden across the disease continuum [11].

Taken together, these factors highlight the complexity of interpreting right ventricular dysfunction in SSc.

The inclusion of both echocardiographic and cardiac magnetic resonance data introduces methodological variability, which should be considered when interpreting the results.

The interpretation of our findings should be considered exploratory, given the limited number of included studies and the substantial heterogeneity across study populations, imaging modalities, and study endpoints.

The included studies encompassed heterogeneous cohorts, including early SSc without pulmonary hypertension, established SSc-associated pulmonary arterial hypertension (SSc-PAH), and mixed populations, as well as different imaging approaches such as speckle-tracking echocardiography and cardiac magnetic resonance.

This heterogeneity is not only methodological but also reflects the intrinsic biological variability of SSc, which may influence both myocardial involvement and cardiopulmonary coupling across different disease phenotypes.

Although subgroup analyses (e.g., early SSc without PH, established SSc-PAH, CMR-based studies, or prognostic versus diagnostic studies) would be of considerable interest, they were not feasible due to the limited number of eligible studies and their methodological variability. Instead, we structured the narrative synthesis to reflect these distinctions, thereby enhancing clarity and interpretability.

### 4.1. Overview of Right Ventricular Dysfunction in SSc

Although SSc has been recognized for more than a century, right ventricular (RV) dysfunction remains one of its most clinically meaningful yet often underappreciated manifestations [21,22]. Even in the absence of overt pulmonary hypertension (PH), subtle abnormalities in RV contractility can be detected in a substantial proportion of patients, reflecting early myocardial fibrosis, microvascular ischemia, or afterload mismatch caused by subclinical pulmonary vascular disease [9,10,12,15]. These early impairments frequently precede symptomatic deterioration, underscoring the importance of timely identification for guiding management and prognostication [27,28].

Conventional echocardiographic parameters—such as tricuspid annular plane systolic excursion (TAPSE), fractional area change (FAC), and tricuspid regurgitant velocity (TRV)—frequently fail to capture these early abnormalities, particularly in patients with preserved hemodynamics or only mild elevations in pulmonary pressure. In contrast, speckle-tracking echocardiography (STE) and cardiac magnetic resonance (CMR) feature-tracking provide a more refined assessment of RV mechanics, enabling evaluation of regional and global myocardial deformation, including basal, mid-ventricular, and apical free-wall strain. Reductions in RV free-wall strain (RV-FWS) and global longitudinal strain (RV-GLS) have been consistently demonstrated even in SSc patients without established PH, highlighting the superior sensitivity of deformation imaging for detecting early myocardial involvement [9,10,12,13].

Restrictive ventilatory defects and parenchymal fibrosis—hallmarks of SSc-related interstitial lung disease (ILD)—further contribute to RV load imbalance and progressive remodeling [27,28]. Unlike other causes of RV dysfunction, where increased afterload is typically the dominant factor, SSc also involves an intrinsic myocardial component, characterized by microvascular rarefaction, collagen deposition, and patchy fibrosis, which preferentially affect longitudinal fibers. This dual mechanistic burden explains why strain abnormalities are often present long before pulmonary artery pressures become significantly elevated [9,12,15].

Similar limitations of conventional parameters have been described in other connective-tissue diseases and vasculopathies. In SSc, these limitations reinforce the need for diagnostic approaches that are less influenced by loading conditions and patient effort—supporting the use of strain-based imaging in combination with circulating biomarkers [9,10,12,13,17,18,19].

Taken together, current evidence suggests that deformation-based RV indices and natriuretic peptides may capture complementary dimensions of cardiopulmonary stress in SSc. However, direct correlations between strain and biomarkers have been reported in only a limited number of studies, underscoring the need for more integrated, multimodal approaches in future research [13,17].

### 4.2. RV Strain and the Limitations of Load-Dependent Assessment

Conventional echocardiographic assessment of right ventricular (RV) systolic performance relies largely on parameters such as tricuspid annular plane systolic excursion (TAPSE), fractional area change (FAC), and tissue Doppler-derived S′ velocity. Although widely used in clinical practice, these indices are highly dependent on loading conditions, cardiac geometry, and tethering effects—factors that are particularly variable in SSc due to the coexistence of ILD, evolving pulmonary hypertension (PH), and intrinsic myocardial fibrosis [21,22,27,28]. Consequently, conventional parameters often underestimate early or subtle RV dysfunction, especially in patients with borderline pulmonary pressures or those categorized within the intermediate-probability zone for PH according to current guideline algorithms.

Load dependency represents a major limitation in this context. Progressive pulmonary fibrosis alters chest wall mechanics, increases RV afterload, and disrupts ventricular–arterial coupling even before overt PH develops [27,28]. These dynamic changes may produce transient shifts in TAPSE or FAC that do not accurately reflect intrinsic myocardial contractility [14,15]. Furthermore, in SSc, myocardial involvement often precedes measurable hemodynamic abnormalities; standard indices therefore fail to capture early microvascular ischemia or patchy fibrosis that selectively impair longitudinal fiber function [9,12,21,22].

Speckle-tracking echocardiography (STE)-derived RV strain provides a more comprehensive assessment of myocardial deformation by quantifying longitudinal fiber shortening—the principal driver of RV contraction [9,10,12,13]. Although RV strain is not completely load-independent, it is substantially less influenced by acute fluctuations in afterload compared with conventional parameters [14,15]. Transient increases in pulmonary vascular resistance, hypoxic vasoconstriction, or ILD-related mechanical changes may modestly impact strain values, but RV free-wall strain (RV-FWS) and global longitudinal strain (RV-GLS) remain markedly more sensitive to intrinsic myocardial dysfunction and more stable across physiological variability [27,28].

Interpretation is further complicated by the heterogeneity of SSc phenotypes. Patients with limited cutaneous disease may exhibit early pulmonary vascular remodeling with disproportionate RV–pulmonary artery (PA) uncoupling, whereas those with diffuse cutaneous involvement more commonly demonstrate ILD-driven afterload excess [8,27,28]. This biological heterogeneity challenges the utility of any single conventional echocardiographic parameter. RV strain offers an important advantage by detecting early dysfunction irrespective of whether the predominant driver is vascular remodeling, fibrotic lung disease, or primary myocardial involvement [9,10,12].

Technical variability across ultrasound vendors, strain algorithms, and acquisition protocols can also affect reproducibility [10]. Nevertheless, most included studies reported acceptable interobserver variability when standardized methodology was applied, reinforcing the feasibility of RV strain assessment in routine practice [9,10,12,13].

In summary, although RV strain retains a degree of load dependency, it appears to offer improved sensitivity compared with traditional echocardiographic measures for detecting early and clinically meaningful RV dysfunction in SSc [14,15]. This is particularly relevant when ILD severity, pulmonary vascular involvement, and biomarker profiles present discordant signals [17,18,19,27,28], positioning RV strain as a key component of comprehensive cardiopulmonary evaluation in SSc.

The clinical differences between conventional echocardiographic parameters and speckle-tracking-derived right ventricular strain across various SSc scenarios are summarized in Table 3.

**Table 3:** Clinical differences between conventional Echocardiography and speckle-tracking.

Study (Year) Clinical Scenario	Conventional Echo Findings	RV Strain Findings	Clinical Interpretation
Osgueritchian et al., 2025—Confirmed SSc-PAH [24]	TAPSE may be mildly reduced; RV size often normal–mildly enlarged; TRV elevated	RV-FWS & RV-GLS ↓; RV–PA coupling impaired	Improves 1-year mortality prediction beyond risk scores
Lui et al., 2025—SSc-PAH (phenotypic spectrum) [25]	Conventional parameters heterogeneous	Distinct LV–RV strain phenotypes associated with outcomes	Identifies high-risk phenotypes
Stronati 2020—SSc without PH [6]	Normal conventional parameters	Progressive RV/LV GLS decline	Detects early myocardial dysfunction
Tennøe 2019—Unselected SSc cohort [1]	TAPSE declines over time; LV EF remains stable; conventional echo often insensitive	RV systolic strain (GLS/FWS) is impaired even when TAPSE only mildly decline	Predicts mortality earlier than conventional indices
Hekimsoy 2018—Early SSc/regional analysis [5]	TAPSE may remain normal	Apical RV-FWS most impaired in PAH; regional strain changes at follow-up	Predicts incident PAH
Vos 2022—CMR-based cohort [7]	Not assessed by echocardiography	RA strain predicts mortality; RV strain less predictive	Highlights role of atrial mechanics
Fernández et al. 2024—SSc cohort (CMR-based) [23]	Not systematically reported	Impaired RV longitudinal strain and reduced RVEF; associated with myocardial fibrosis (LGE)	Suggests increased risk of heart failure hospitalization and mortality; supports association between myocardial fibrosis and RV dysfunction

Abbreviations: SSc, systemic sclerosis; PH, pulmonary hypertension; PAH, pulmonary arterial hypertension; CMR, cardiac magnetic resonance; RV, right ventricle; LV, left ventricle; RA, right atrium; RV-FWS, right ventricular free-wall strain; RV-GLS, right ventricular global longitudinal strain; LV GLS, left ventricular global longitudinal strain; TRV, tricuspid regurgitant velocity; TAPSE, tricuspid annular plane systolic excursion.

### 4.3. Early RV Involvement in SSc and Limitations of Conventional Echocardiography

Across multiple studies using speckle-tracking echocardiography, right ventricular (RV) free-wall longitudinal strain (RV-FWS) and global longitudinal strain (RV-GLS) were frequently reduced even when conventional echocardiographic markers—such as tricuspid annular plane systolic excursion (TAPSE), fractional area change (FAC), RV size, or tricuspid regurgitation velocity (TRV)—remained within normal limits. This dissociation was observed across both early-stage disease and established SSc cohorts.

In asymptomatic SSc patients without PH, Hekimsoy et al. and Stronati et al. demonstrated regionally impaired RV-FWS or progressively worsening GLS despite preserved TAPSE and normal chamber dimensions [5,6]. Similarly, Tennøe et al. (2019) showed that over time, TAPSE decline was detectable but lagged behind strain impairment, suggesting that deformation abnormalities precede overt RV systolic dysfunction detectable by standard echocardiography [1].

Collectively, these findings support the concept that microvascular dysfunction, subclinical myocardial fibrosis, and subtle afterload elevation affect the RV early in the disease course, and that deformation imaging is better suited than conventional metrics to detect these early impairments

### 4.4. RV Strain as a Marker of Disease Progression and Clinical Outcomes in SSc

Progressive worsening of RV strain was commonly observed in studies with longitudinal assessment. Over 12 months, Hekimsoy et al. observed deterioration in regional RV-FWS, while Stronati et al. reported significant decline in both LV and RV GLS over 20 months. These longitudinal changes correlated with subsequent development of PH and a higher incidence of major adverse cardiac events (MACE), highlighting the prognostic importance of early deformation abnormalities [5,6].

Mortality associations were reported in two large cohorts. Tennøe et al. (2019) showed that RV systolic dysfunction—captured by both TAPSE decline and abnormal strain—was associated with increased all-cause mortality [1]. Similarly, Lui et al. (2025) identified strain-derived phenotypes associated with worse survival in SSc-PAH, further supporting the potential prognostic value of deformation metrics in advanced disease [25].

These findings suggest that RV strain may have clinical relevance not only as a diagnostic tool but also as an indicator of disease progression and long-term outcomes.

### 4.5. RV–Pulmonary Artery Coupling and Prognostic Refinement

Strain-derived RV–PA coupling has emerged as an important indicator of the interplay between intrinsic RV contractility and pulmonary vascular load. In RHC-confirmed SSc-PAH, Osgueritchian et al. demonstrated that RV-FWS/PASP and RV-GLS/PASP significantly improved discrimination for 1-year mortality when integrated into established prognostic models such as REVEAL, COMPERA, and the French non-invasive criteria. These findings suggest that strain-derived coupling metrics capture subtle impairments in RV adaptation that are not reflected in conventional indices or risk scores [24].

Although it did not meet the inclusion criteria for the final synthesis due to the absence of RV strain assessment, the study by Grimaldi et al. provides complementary biological context. It showed that TAPSE/sPAP—a simplified surrogate of RV–PA coupling—correlated with elevated natriuretic peptide levels (NT-proANP) and improved early identification of patients at increased risk for pulmonary vascular involvement compared with TRV alone [26]. These findings reinforce the concept that biomarker–based evaluation may enhance the interpretation of RV–PA coupling, supporting a more integrated approach to risk stratification across the SSc spectrum.

### 4.6. Complementary Insights from CMR-Derived Deformation Analysis

The two CMR-based studies included in this review add important complementary evidence. Vos et al. demonstrated that RA reservoir and conduit strain were independent predictors of mortality, even when RV strain was not. This underscores the importance of atrial mechanics in capturing right-sided hemodynamic stress [7].

Fernández et al. further showed that RV dysfunction (RVD) identified by CMR—defined by reduced RVEF and impaired RV longitudinal strain—was associated with higher BNP levels, greater late gadolinium enhancement (LGE), and increased risk of heart failure hospitalization and mortality. Notably, reduced DLCO and pulmonary artery trunk dilation were independent predictors of RVD, reinforcing the interdependence between pulmonary and cardiac involvement in SSc [23].

These findings suggest that CMR feature tracking may provide additional value for characterizing tissue-level changes and functional impairment when echocardiographic windows are suboptimal or when fibrosis is suspected.

### 4.7. Role of Biomarkers in RV Dysfunction Assessment

Despite the clinical relevance of natriuretic peptides in cardiopulmonary disease, biomarker integration in RV strain studies has been limited. Although excluded from the quantitative synthesis due to the absence of speckle-tracking-derived RV strain assessment, the study by Grimaldi et al. [26] provides important biological context relevant to this review. Their findings demonstrated that elevations in NT-proANP were associated with impaired TAPSE/sPAP, a surrogate of RV–PA coupling, and improved early identification of patients at higher risk for pulmonary hypertension. This finding suggests a potential complementary role of natriuretic peptides in reflecting myocardial stress and pulmonary vascular load, supporting the potential clinical value of integrating biomarker information with deformation-based imaging in SSc [26].

Although this provides preliminary evidence that biomarkers may complement RV deformation analysis, the lack of consistent BNP or NT-proBNP assessment across studies precludes firm conclusions. Future standardized studies integrating both imaging and biomarker assessment will be essential to better define their combined diagnostic and prognostic value in SSc.

### 4.8. Limitations of the Included Studies

Across the seven included studies, several methodological limitations should be acknowledged when interpreting the findings of this review. The majority of investigations were observational—either retrospective or prospective cohorts—which introduces an inherent risk of confounding. Key clinical variables relevant to SSc, such as disease duration, autoantibody profile, severity of interstitial lung disease (ILD), comorbid cardiopulmonary conditions, and background therapy, were inconsistently reported and only partially adjusted for in multivariable analyses. This incomplete adjustment limits the ability to determine whether strain abnormalities reflect intrinsic myocardial impairment, pulmonary vascular load, or a combination of both.

Selection bias was also frequently observed. Most cohorts were derived from tertiary referral centers where patients often present with more advanced disease or unexplained cardiopulmonary symptoms. The two cardiac magnetic resonance (CMR) studies (Vos and Fernández) included only those referred for advanced imaging, likely enriching the sample with individuals suspected of cardiac involvement [7,23]. Similarly, the SSc-PAH cohorts (Lui, Osgueritchian) consisted exclusively of right-heart-catheterization-confirmed cases, which may not represent the broader SSc population [24,25]. As a result, the generalizability of these findings to early or mild disease remains limited.

Outcome measurement was generally robust in studies evaluating mortality or major clinical events; however, several investigations lacked standardized follow-up protocols or clear definitions for incident pulmonary hypertension (PH). Longitudinal assessments were available in only a subset of studies—most notably Hekimsoy and Stronati—limiting the capacity to evaluate trajectories of right ventricular (RV) strain or RV–pulmonary artery (PA) coupling over time [5,6].

Missing data represented a recurrent limitation. Many studies experienced exclusions due to suboptimal acoustic windows, incomplete biomarker data, or unavailable follow-up information. In some cases, analytic samples were substantially smaller than the initial cohorts, and missingness was not systematically addressed through imputation or sensitivity analyses, raising concerns regarding potential bias.

Technical variability further contributes to heterogeneity across studies. Differences in ultrasound vendors, strain software, feasibility thresholds, and region-of-interest definition may have influenced measured RV free-wall strain (RV-FWS) and RV global longitudinal strain (RV-GLS) values, reducing comparability. While several studies reported acceptable interobserver variability when standardized acquisition protocols were followed, reproducibility was not uniformly assessed. For CMR feature tracking, variability in post-processing algorithms and strain extraction methods may similarly affect direct comparison between studies.

Finally, biomarker integration was limited across the available literature. Only one study (Grimaldi et al.) systematically evaluated natriuretic peptides; however, because it did not include RV strain assessment, it was not eligible for inclusion in the final synthesis [26]. Its findings were restricted to the relationship between NT-proANP and TAPSE/sPAP, a simplified surrogate of RV–PA coupling, rather than deformation-based metrics. The absence of consistent BNP or NT-proBNP reporting in studies that did evaluate RV strain limits the ability to determine the combined diagnostic or prognostic value of biomarkers and advanced deformation parameters.

Taken together, these limitations highlight the need for larger, multicenter, prospective studies with standardized imaging protocols, comprehensive biomarker assessment, and rigorous adjustment for disease heterogeneity. Such efforts will be important to better define the role of RV strain and RV–PA coupling in the cardiopulmonary evaluation of patients with SSc.

### 4.9. Implications for Future Research

The findings of this review highlight several important directions for future research aimed at improving the cardiopulmonary evaluation of patients with SSc. First, there is a clear need for larger, prospective, multicenter studies using standardized acquisition and post-processing protocols for right ventricular (RV) strain and RV–pulmonary artery (PA) coupling assessment, given the heterogeneity arising from variable strain algorithms, vendor differences, and inconsistent feasibility criteria [11].

Second, longitudinal investigations are essential to determine how early RV strain abnormalities evolve over time and how they relate to clinically meaningful outcomes such as incident pulmonary hypertension (PH), worsening interstitial lung disease (ILD), right-heart failure, and mortality. Only two of the included studies incorporated serial imaging, and both demonstrated progressive deterioration of deformation parameters despite relatively stable conventional indices [9,12]—supporting the value of systematic longitudinal assessment in clarifying the temporal interplay among myocardial involvement, pulmonary vascular remodeling, and ILD progression [27,28].

Third, the integration of imaging and circulating biomarkers remains insufficiently explored. Across the eight included studies, only one evaluated natriuretic peptides in conjunction with advanced cardiac imaging, and none assessed direct correlations between NT-proBNP and RV strain. Given the continuum linking myocardial stress, vascular load, and neurohormonal activation in SSc, future research should prioritize multimodal approaches that incorporate strain, coupling indices, tissue characterization, DLCO, biomarkers, and functional capacity within the same analytic framework [8,15,17,18,19]. Studies such as Grimaldi et al., although not fulfilling strain-specific inclusion criteria, illustrate how natriuretic peptides may enhance risk stratification when combined with RV–PA coupling indices, supporting future protocols that integrate biomarkers with advanced deformation imaging [26].

Fourth, right atrial (RA) mechanics and CMR-derived parameters warrant further study. The prognostic relevance of RA strain and CMR evidence of fibrosis suggests that atrial–ventricular interplay and myocardial tissue characterization may improve early risk stratification beyond RV strain alone. Comparative studies assessing the incremental value of CMR versus echocardiographic strain, particularly in patients with suboptimal acoustic windows, are needed [7,16].

Finally, future work should aim to better define clinically meaningful thresholds for RV strain and strain-derived coupling indices specific to SSc. Although abnormal values were consistently associated with adverse outcomes, no study established validated cut-offs for early disease detection or prognostic stratification [8,14]. Establishing such thresholds would facilitate the incorporation of deformation imaging into routine clinical pathways and inform targeted surveillance strategies for high-risk SSc phenotypes.

Overall, future research integrating standardized deformation imaging, RV–PA coupling, CMR characterization, and comprehensive biomarker evaluation will be important to advance precision cardiopulmonary phenotyping and improve early detection and risk stratification in SSc [11].

## 5. Conclusions

This systematic review suggests that right ventricular (RV) deformation imaging may provide meaningful diagnostic and prognostic insight in SSc. Across heterogeneous cohorts—including patients without pulmonary hypertension (PH), unselected SSc populations, and individuals with confirmed SSc–associated pulmonary arterial hypertension (SSc-PAH)—RV free-wall strain (RV-FWS), RV strain and RV–PA coupling indices appear to offer improved sensitivity compared with conventional echocardiographic parameters for detecting early RV dysfunction and may provide additional prognostic information in SSc.

However, current evidence remains limited by small study numbers, methodological heterogeneity, and inconsistent biomarker integration.

Standardized prospective studies incorporating both deformation imaging and biomarkers are needed to better define their diagnostic and prognostic role in clinical practice.

These findings may reflect a particular susceptibility of the RV in SSc, potentially related to the interplay between microvascular dysfunction, myocardial fibrosis, and evolving pulmonary vascular load.

Cardiac magnetic resonance (CMR) feature tracking may further complement echocardiographic assessment by offering additional information on RV mechanics, right atrial (RA) function, and myocardial tissue characteristics, which in several studies were associated with adverse outcomes. Biomarker data remain limited; however, natriuretic peptides may provide complementary information regarding cardiopulmonary burden, although current evidence does not support definitive conclusions.

Overall, the current literature indicates that RV strain and strain-derived coupling indices may represent promising tools for refining cardiopulmonary evaluation in SSc, particularly in patients with early symptoms or borderline hemodynamic findings. However, the available evidence is limited by small study numbers, substantial methodological heterogeneity, and restricted longitudinal data. Future standardized, prospective studies integrating deformation imaging and biomarkers are needed before these parameters can be fully incorporated into routine clinical algorithms.

## Figures and Tables

**Table 1:** Summary of database search strategy.

Database	Search String Used	Results (*n*)
PubMed	(“systemic sclerosis” OR “scleroderma”) AND (“right ventricular strain” OR “RV strain” OR “speckle tracking”) AND (“pulmonary hypertension” OR “interstitial lung disease” OR “NT-proBNP”)	18
Web of Science		78
ScienceDirect		338

## Data Availability

Data are contained within the article.

## Supplementary Materials

The following supporting information can be downloaded at https://www.mdpi.com/article/10.3390/jcm15093368/s1, Table S1: PRISMA checklist.
