# Supplementary material for: Right Ventricular Strain and RV–Pulmonary Artery Coupling in Systemic Sclerosis: A Systematic Review
Source: J Clin Med. 2026 Apr 28;15(9):3368. doi: 10.3390/jcm15093368 (PMC13163976; doi:10.3390/jcm15093368)
Supplement: Supplementary file 1 [file jcm-15-03368-s001.zip › jcm-4243907-supplementary.pdf]

# PRISMA 2020 Checklist

| Section and Topic | Item # | Checklist item | Location where item is reported |
|---|---|---|---|
| **TITLE** | | | |
| Title | 1 | Identify the report as a systematic review. | Title |
| **ABSTRACT** | | | |
| Abstract | 2 | See the PRISMA 2020 for Abstracts checklist. | Abstract |
| **INTRODUCTION** | | | |
| Rationale | 3 | Describe the rationale for the review in the context of existing knowledge. | Introduction – paragraphs 1–3 |
| Objectives | 4 | Provide an explicit statement of the objective(s) or question(s) the review addresses. | Introduction – Objectives section |
| **METHODS** | | | |
| Eligibility criteria | 5 | Specify the inclusion and exclusion criteria for the review and how studies were grouped for the syntheses. | Methods – Eligibility criteria |
| Information sources | 6 | Specify all databases, registers, websites, organisations, reference lists and other sources searched or consulted to identify studies. Specify the date when each source was last searched or consulted. | Methods – Information sources |
| Search strategy | 7 | Present the full search strategies for all databases, registers and websites, including any filters and limits used. | Methods – Search strategy |
| Selection process | 8 | Specify the methods used to decide whether a study met the inclusion criteria of the review, including how many reviewers screened each record and each report retrieved, whether they worked independently, and if applicable, details of automation tools used in the process. | Methods – Study selection |
| Data collection process | 9 | Specify the methods used to collect data from reports, including how many reviewers collected data from each report, whether they worked independently, any processes for obtaining or confirming data from study investigators, and if applicable, details of automation tools used in the process. | Methods – Data extraction |
| Data items | 10a | List and define all outcomes for which data were sought. Specify whether all results that were compatible with each outcome domain in each study were sought (e.g. for all measures, time points, analyses), and if not, the methods used to decide which results to collect. | Methods – Outcomes |
| | 10b | List and define all other variables for which data were sought (e.g. participant and intervention characteristics, funding sources). Describe any assumptions made about any missing or unclear information. | Methods – Data extraction |
| Study risk of bias assessment | 11 | Specify the methods used to assess risk of bias in the included studies, including details of the tool(s) used, how many reviewers assessed each study and whether they worked independently, and if applicable, details of automation tools used in the process. | Methods – Risk of bias |
| Effect measures | 12 | Specify for each outcome the effect measure(s) (e.g. risk ratio, mean difference) used in the synthesis or presentation of results. | Not applicable – narrative synthesis |
| Synthesis methods | 13a | Describe the processes used to decide which studies were eligible for each synthesis (e.g. tabulating the study intervention characteristics and comparing against the planned groups for each synthesis (item #5)). | Methods – Data synthesis |
| | 13b | Describe any methods required to prepare the data for presentation or synthesis, such as handling of missing summary statistics, or data conversions. | Methods – Data synthesis |
| | 13c | Describe any methods used to tabulate or visually display results of individual studies and syntheses. | Methods – Data synthesis |
| | 13d | Describe any methods used to synthesize results and provide a rationale for the choice(s). If meta-analysis was performed, describe the model(s), method(s) to identify the presence and extent of statistical heterogeneity, and software package(s) used. | Methods – Data synthesis |

# PRISMA 2020 Checklist

| Section and Topic | Item # | Checklist item | Location where item is reported |
|---|---|---|---|
| | 13e | Describe any methods used to explore possible causes of heterogeneity among study results (e.g. subgroup analysis, meta-regression). | Not applicable – no statistical methods |
| | 13f | Describe any sensitivity analyses conducted to assess robustness of the synthesized results. | Not applicable – no sensitivity analyses conducted. |
| Reporting bias assessment | 14 | Describe any methods used to assess risk of bias due to missing results in a synthesis (arising from reporting biases). | Methods – Risk of bias |
| Certainty assessment | 15 | Describe any methods used to assess certainty (or confidence) in the body of evidence for an outcome. | Methods – Certainty of evidence assessment |
| **RESULTS** | | | |
| Study selection | 16a | Describe the results of the search and selection process, from the number of records identified in the search to the number of studies included in the review, ideally using a flow diagram. | Results – Study selection (Figure 1, PRISMA flow diagram) |
| | 16b | Cite studies that might appear to meet the inclusion criteria, but which were excluded, and explain why they were excluded. | Results – Study selection (excluded studies described in text) |
| Study characteristics | 17 | Cite each included study and present its characteristics. | Results – Study characteristics (Table 2) |
| Risk of bias in studies | 18 | Present assessments of risk of bias for each included study. | Results – Risk of bias (Section 3.3, Figure 2, Supplementary Table S2) |
| Results of individual studies | 19 | For all outcomes, present, for each study: (a) summary statistics for each group (where appropriate) and (b) an effect estimate and its precision (e.g. confidence/credible interval), ideally using structured tables or plots. | Results – Table 2 (summary of individual study findings) |
| Results of syntheses | 20a | For each synthesis, briefly summarise the characteristics and risk of bias among contributing studies. | Results – Synthesis of results (Sections 3.2 and 3.3) |
| | 20b | Present results of all statistical syntheses conducted. If meta-analysis was done, present for each the summary estimate and its precision (e.g. confidence/credible interval) and measures of statistical heterogeneity. If comparing groups, describe the direction of the effect. | Not applicable – no statistical synthesis or meta-analysis was conducted |
| | 20c | Present results of all investigations of possible causes of heterogeneity among study results. | Not applicable – no meta-analysis performed. |
| | 20d | Present results of all sensitivity analyses conducted to assess the robustness of the synthesized results. | Not applicable – no meta-analysis performed. |
| Reporting biases | 21 | Present assessments of risk of bias due to missing results (arising from reporting biases) for each synthesis assessed. | Not applicable – no meta-analysis performed. |

| Section and Topic | Item # | Checklist item | Location where item is reported |
|---|---|---|---|
| Certainty of evidence | 22 | Present assessments of certainty (or confidence) in the body of evidence for each outcome assessed. | Certainty of evidence was assessed qualitatively and considered moderate, limited by observational design, heterogeneity, and risk of bias.ongoing |
| **DISCUSSION** | | | |
| Discussion | 23a | Provide a general interpretation of the results in the context of other evidence. | The results of this review are consistent with previous evidence showing that RV dysfunction is a key determinant of prognosis in systemic sclerosis and that conventional echocardiographic parameters are often insufficient for early detection. In agreement with prior studies, RV strain and RV–PA coupling indices demonstrated superior sensitivity and prognostic value. These findings further support the integration of advanced deformation imaging into the assessment of cardiopulmonary involvement in SSc. |
| | 23b | Discuss any limitations of the evidence included in the review. | The evidence included in this review is limited by observational study designs, risk of confounding, substantial heterogeneity in populations and methodologies, and potential selection and reporting biases. Small sample sizes, limited longitudinal data, and technical variability in strain assessment further reduce the strength and generalizability of the findings. |
| | 23c | Discuss any limitations of the review processes used. | Limitations of the review process include restriction to English-language full-text |

# PRISMA 2020 Checklist

| Section and Topic | Item # | Checklist item | Location where item is reported |
|---|---|---|---|
| | | | studies, potential reviewer bias during study selection, absence of meta-analysis and formal bias assessments, lack of GRADE evaluation, and reliance on published data only. |
| | 23d | Discuss implications of the results for practice, policy, and future research. | These findings support the integration of RV strain and RV–PA coupling into the clinical assessment of systemic sclerosis, particularly for early detection and risk stratification. Future guidelines may incorporate these parameters; however, standardized protocols and validated thresholds are required. Further large, prospective, and multimodal studies are needed to confirm their clinical utility. |
| **OTHER INFORMATION** | | | |
| Registration and protocol | 24a | Provide registration information for the review, including register name and registration number, or state that the review was not registered. | This systematic review was prospectively registered in the International Prospective Register of Systematic Reviews (PROSPERO) under the registration number **CRD420251235679** |
| | 24b | Indicate where the review protocol can be accessed, or state that a protocol was not prepared. | Available in PROSPERO (CRD420251235679) |
| | 24c | Describe and explain any amendments to information provided at registration or in the protocol. | No amendments were made to the protocol or the information provided at the time of registration in the PROSPERO database. |
| Support | 25 | Describe sources of financial or non-financial support for the review, and the role of the funders or sponsors in the review. | The authors received non-financial support through the AdAugusta research career counseling program (Project code: |

# PRISMA 2020 Checklist

| Section and Topic | Item # | Checklist item | Location where item is reported |
|---|---|---|---|
| | | | 2/16.11.2022; Financing contract: DGGC PNRR No. 760030/23.05.2023).<br>The funders had no role in the design, conduct, analysis, or reporting of the review. |
| Competing interests | 26 | Declare any competing interests of review authors. | The authors declare no competing interests. |
| Availability of data, code and other materials | 27 | Report which of the following are publicly available and where they can be found: template data collection forms; data extracted from included studies; data used for all analyses; analytic code; any other materials used in the review. | Data available on request; no analytic code generated. |

*From:* Page MJ, McKenzie JE, Bossuyt PM, Boutron I, Hoffmann TC, Mulrow CD, et al. The PRISMA 2020 statement: an updated guideline for reporting systematic reviews. BMJ 2021;372:n71. doi: 10.1136/bmj.n71.
This work is licensed under CC BY 4.0. To view a copy of this license, visit https://creativecommons.org/licenses/by/4.0/
